# RETRACTION: Thyroxine Alleviates Energy Failure, Prevents Myocardial Cell Apoptosis, and Protects against Doxorubicin‐Induced Cardiac Injury and Cardiac Dysfunction via the LKB1/AMPK/mTOR Axis in Mice

**DOI:** 10.1155/dim/9851484

**Published:** 2026-05-21

**Authors:** Disease Markers

RETRACTION: Y. Wang, S. Zhu, H. Liu, et al., “Thyroxine Alleviates Energy Failure, Prevents Myocardial Cell Apoptosis, and Protects against Doxorubicin‐Induced Cardiac Injury and Cardiac Dysfunction via the LKB1/AMPK/mTOR Axis in Mice,” *Disease Markers* 2019, no. 1 (2019): 7420196, https://doi.org/10.1155/2019/7420196.

The above article, published online on 18 December 2019 in Wiley Online Library (wileyonlinelibrary.com), has been retracted by John Wiley & Sons Ltd. The retraction has been agreed after an investigation into image concerns raised by *Aneurus inconstans* via PubPeer [1].

Specifically, the blot representing p‐AMPK in Figure 6c shares unexpected similarities with the blot representing Bax in Figure 6c of another article authored by the same group [2].

Repeated attempts by the publisher to contact the authors have not resulted in a reply. As such, the results and conclusions cannot be considered reliable. The article is therefore retracted.
